# Supplementary material for: SARS-CoV-2 antibody prevalence in England following the first peak of the pandemic
Source: Nat Commun. 2021 Feb 10;12:905. doi: 10.1038/s41467-021-21237-w (PMC7876103; doi:10.1038/s41467-021-21237-w)

Supplementary Information

Ward H, Atchison C, Whitaker M et al. December 2020

Contents

Supplementary Table 1: Prevalence of SARS-CoV-2 IgG antibodies, adults in England, by sociodemographic characteristics 2

Supplementary Table 2: Prevalence of SARS-CoV-2 IgG antibodies, adults in England, by detailed ethnicity categories 3

Supplementary Figure 1: Logistic regression for SARS-CoV-2 antibodies: adjusted Odds Ratios (95% CI) for sociodemographic and clinical covariates 4

Supplementary Table 3: Prevalence of SARS-CoV-2 IgG antibodies, adults in England, by individual and clinical characteristics 6

Supplementary Table 4: Prevalence of SARS-CoV-2 IgG antibodies, adults in England, by household income and age 8

Supplementary Table 5 Infection Fatality Ratio sensitivity analyses including care home residents and all excess deaths 9

Supplementary Methods 10

Weighting strategy 10

Sample profile 10

Sampling frame, response and weighting 11

Study response rates 11

Regions of England 12

Selected characteristics of Regions of England 12

Map showing Regions of England with prevalence of SARS-CoV-2 antibody 13

Missing data 14

Supplementary Table 1: Prevalence of SARS-CoV-2 IgG antibodies, adults in England, by sociodemographic characteristics

	**Total tests** with valid results	***Total antibody positive***	**Crude prevalence %** [95% confidence intervals]	**Prevalence % adjusted for test*** [95% confidence intervals]	**Prevalence % adjusted* and weighted**† [95% confidence intervals]
England	99908	5544	5.6 [5.4-5.7]	5.0 [4.8-5.2]	6.0 [5.8-6.1]
**Sex**					
Male	43825	2405	5.5 [5.3-5.7]	4.9 [4.7-5.2]	6.2 [5.9-6.4]
Female	56083	3139	5.6 [5.4-5.8]	5.1 [4.8-5.3]	5.8 [5.5-6.0]
**Age**					
18-24	6499	463	7.1 [6.5-7.8]	6.9 [6.2-7.7]	7.9 [7.3-8.5]
25-34	13366	930	7.0 [6.5-7.4]	6.7 [6.2-7.2]	7.8 [7.4-8.3]
35-44	17052	964	5.7 [5.3-6.0]	5.1 [4.7-5.6]	6.1 [5.7-6.6]
45-54	20634	1255	6.1 [5.8-6.4]	5.6 [5.3-6.0]	6.4 [6.0-6.9]
55-64	20404	1131	5.5 [5.2-5.9]	5.0 [4.6-5.4]	5.9 [5.5-6.4]
65-74	15543	568	3.7 [3.4-4.0]	2.7 [2.4-3.1]	3.2 [2.8-3.6]
75+	6410	233	3.6 [3.2-4.1]	2.7 [2.2-3.3]	3.3 [2.9-3.8]
**Ethnicity**					
White	92737	4827	5.2 [5.1-5.3]	4.6 [4.4-4.8]	5.0 [4.8-5.2]
Mixed	1347	106	7.9 [6.5-9.4]	7.8 [6.2-9.7]	8.9 [7.1-11.1]
Asian‡	3658	369	10.1 [9.2-11.1]	10.5 [9.3-11.7]	11.9 [11.0-12.8]
Black§	900	135	15.0 [12.8-17.5]	16.4 [13.8-19.4]	17.3 [15.8-19.0]
Other	762	79	10.4 [8.4-12.7]	10.8 [8.4-13.7]	12.3 [10.2-14.7]
**Deprivation Quintile**‖					
1 most deprived	10082	682	6.8 [6.3-7.3]	6.5 [5.9-7.1]	7.3 [6.8-7.7]
2	16015	947	5.9 [5.6-6.3]	5.4 [5.0-5.9]	6.4 [6.0-6.8]
3	21474	1196	5.6 [5.3-5.9]	5.0 [4.7-5.4]	5.9 [5.5-6.3]
4	24840	1287	5.2 [4.9-5.5]	4.6 [4.2-4.9]	5.2 [4.8-5.6]
5 least deprived	27497	1432	5.2 [5.0-5.5]	4.6 [4.3-4.9]	5.0 [4.6-5.4]
**Household size**					
1	15052	720	4.8 [4.5-5.1]	4.1 [3.7-4.5]	4.7 [4.3-5.1]
2	36413	1784	4.9 [4.7-5.1]	4.2 [4.0-4.5]	5.0 [4.7-5.3]
3	19734	1158	5.9 [5.5-6.2]	5.4 [5.0-5.8]	6.5 [6.0-6.9]
4	19611	1204	6.1 [5.8-6.5]	5.7 [5.3-6.1]	6.4 [6.0-6.8]
5	6403	447	7.0 [6.4-7.6]	6.7 [6.0-7.5]	7.7 [7.0-8.5]
6	1848	152	8.2 [7.1-9.6]	8.2 [6.8-9.8]	12.3 [10.8-14.0]
7+	827	79	9.6 [7.7-11.7]	9.8 [7.6-12.5]	13.0 [11.0-15.3]
**Region**					
North East	3574	196	5.5 [4.8-6.3]	4.9 [4.1-5.9]	5.0 [4.3-5.9]
North West	11996	714	6.0 [5.5-6.4]	5.5 [5.0-6.0]	6.6 [6.1-7.2]
Yorkshire	6519	284	4.4 [3.9-4.9]	3.6 [3.0-4.2]	3.9 [3.5-4.5]
East Midlands	12684	601	4.7 [4.4-5.1]	4.0 [3.6-4.5]	4.2 [3.7-4.8]
West Midlands	9620	547	5.7 [5.2-6.2]	5.2 [4.6-5.7]	5.8 [5.3-6.4]
East of England	14433	805	5.6 [5.2-6.0]	5.0 [4.6-5.5]	5.1 [4.6-5.6]
London	9547	1045	10.9 [10.3-11.6]	11.5 [10.8-12.3]	13.0 [12.3-13.6]
South East	21979	995	4.5 [4.3-4.8]	3.8 [3.4-4.1]	3.9 [3.5-4.3]
South West	9556	357	3.7 [3.4-4.1]	2.8 [2.4-3.3]	2.8 [2.4-3.3]

*All estimates of prevalence adjusted for imperfect test sensitivity and specificity (see methods for details).

†Responses have been re-weighted to account for sample design and for variation in response rate (see methods for details).

‡Asian / Asian British

§ Black / African / Caribbean / Black British

‖Based on Index of Multiple Deprivation (2019) at lower super output area

Supplementary Table 2: Prevalence of SARS-CoV-2 IgG antibodies, adults in England, by detailed ethnicity categories

**Category**	**Total antibody positive**	**Total tests (with valid results)**	**Prevalence % adjusted for test** [95% confidence intervals]
**White**			
English / Welsh / Scottish / Northern Irish / British	4415	86790	4.44 [4.27-4.62]
Irish	78	968	8.02 [6.15-10.29]
Gypsy or Irish traveler	<5*	<50*	n/a
Any other White background	333	4947	6.42 [5.62-7.31]
**Mixed**			
White / Black Caribbean	27	321	8.45 [5.36-12.72]
White / Black African	12	107	11.83 [6.18-20.71]
White and Asian	26	396	6.22 [3.76-9.69]
Any other Mixed / Multiple ethnic background	41	523	7.76 [5.34-10.92]
**Asian / Asian British**			
Indian	177	1847	9.86 [8.34-11.58]
Pakistani	54	503	11.25 [8.34-14.87]
Bangladeshi	24	189	13.61 [8.78-20.24]
Chinese	29	410	6.84 [4.30-10.33]
Other Asian	85	709	12.76 [10.12-15.89]
**Black / African / Caribbean / Black British**			
African	90	519	19.21 [15.57-23.42]
Caribbean	38	320	12.62 [8.89-17.45]
Other Black / African / Caribbean background	7	61	12.14 [5.15-24.63]
**Other ethnic group**			
Arab	15	168	9.07 [4.92-15.43]
Any other ethnic group	64	594	11.29 [8.59-14.61]
Prefer not to say	28	479	5.36 [3.22-8.34]

*suppressed due to small numbers

Supplementary Figure 1: Logistic regression for SARS-CoV-2 antibodies: adjusted Odds Ratios (95% CI) for sociodemographic and clinical covariates

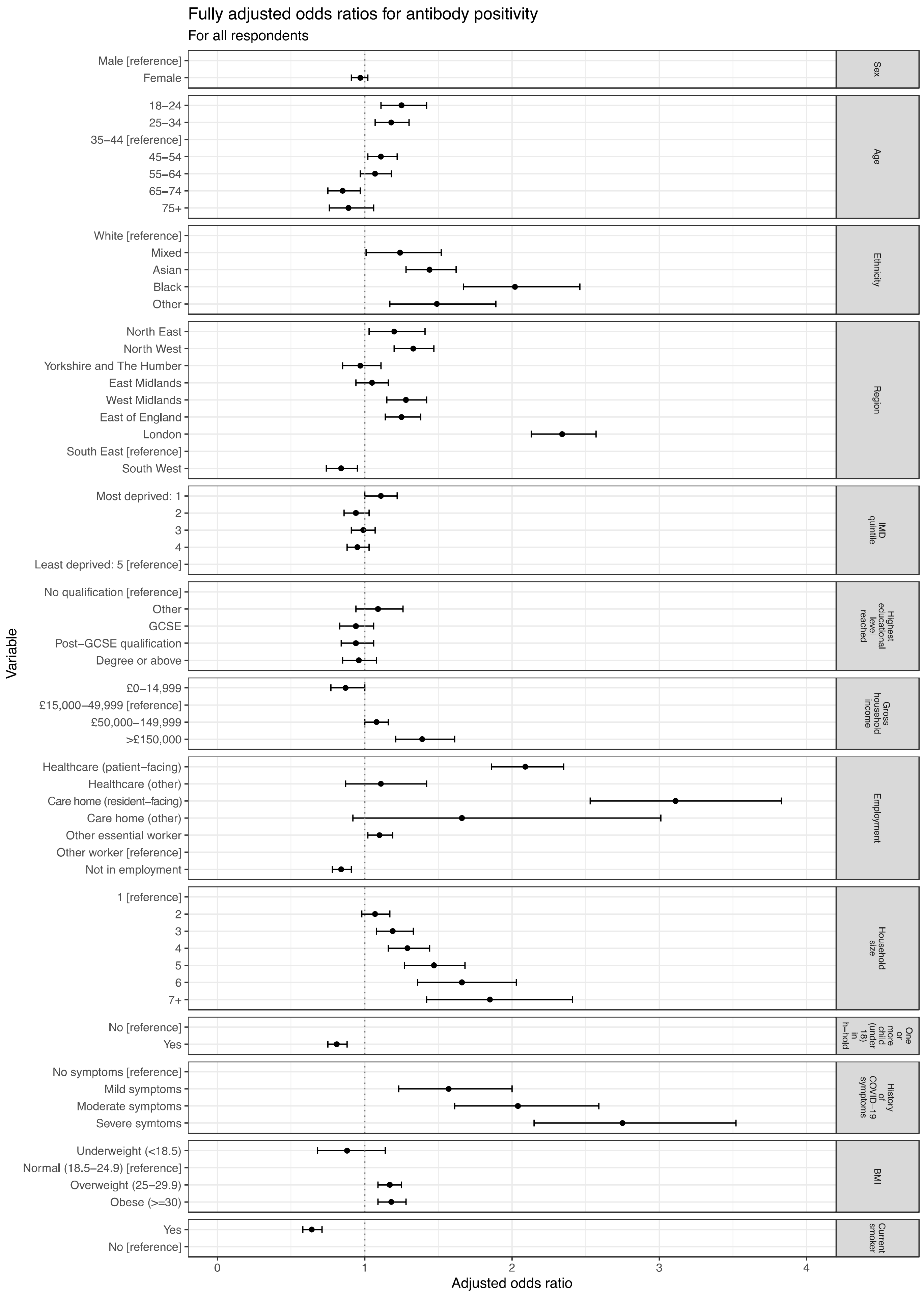

Legend: Jointly adjusted odds ratios [95% confidence intervals] from multivariable logistic regression for the covariate of interest. Jointly adjusted odds ratios were obtained from multivariable logistic regression adjusting for age, sex, ethnicity, region, index of multiple deprivation quintile, education, household income, employment, household size and child in house (y/n). For data see Table 2 and Supplementary Data 1.

Supplementary Table 3: Prevalence of SARS-CoV-2 IgG antibodies, adults in England, by individual and clinical characteristics

	**Total tests** (with valid results)	***Total antibody positive***	**Crude prevalence %** [95% confidence intervals]	**Prevalence % adjusted for test*** [95% confidence intervals]
**Employment**				
Health care (patient-facing)	3402	379	11.1 [10.1-12.2]	11.7 [10.5-13.1]
Health care (other)	1151	73	6.3 [5.1-7.9]	6.0 [4.4-7.8]
Care home (client-facing)	761	115	15.1 [12.7-17.8]	16.5 [13.7-19.8]
Care home (other)	146	12	8.2 [4.8-13.8]	8.2 [4.1-15.0]
Other essential worker[†]	19927	1209	6.1 [5.7-6.4]	5.6 [5.2-6.0]
Other worker[§]	37855	2189	5.8 [5.6-6.0]	5.3 [5.0-5.6]
Not in employment	35737	1516	4.2 [4.0-4.5]	3.4 [3.2-3.7]
**Maximum educational level**				
Degree or above	9101	420	4.6 [4.2-5.1]	3.9 [3.4-4.4]
Post-GCSE qualification	6758	382	5.7 [5.1-6.2]	5.1 [4.5-5.8]
GCSE	22204	1135	5.1 [4.8-5.4]	4.5 [4.1-4.8]
Other	27597	1515	5.5 [5.2-5.8]	4.9 [4.6-5.3]
No qualification	33751	2062	6.1 [5.9-6.4]	5.7 [5.4-6.0]
**Gross Household income**				
£0-14,999	297	6482	4.6 [4.1-5.1]	3.8 [3.2-4.5]
£15,000-49,999	1650	31146	5.3 [5.0-5.6]	4.7 [4.4-5.0]
£50,000-149,999	1707	27252	6.3 [6.0-6.6]	5.9 [5.5-6.2]
>£150,000	284	3216	8.8 [7.9-9.9]	8.9 [7.8-10.2]
**Number children in household**				
0	67515	3647	5.4 [5.2-5.6]	4.8 [4.6-5.0]
1	14471	851	5.9 [5.5-6.3]	5.4 [5.0-5.9]
2	13803	808	5.8 [5.5-6.3]	5.4 [4.9-5.8]
>2	4099	238	5.8 [5.1-6.6]	5.3 [4.5-6.2]
**History of COVID-19**				
Positive PCR test	341	277	81.2 [76.7-85.0]	96.2 [90.8-100.0]
Suspected by doctor	1144	353	30.9 [28.2-33.6]	35.5 [32.3-38.8]
Suspected by respondent	17893	3118	17.4 [16.9-18.0]	19.3 [18.6-20.0]
No	80390	1698	2.1 [2.0-2.2]	0.9 [0.7-1.0]
**Symptoms**				
Appetite loss	5895	1504	25.5 [24.4-26.6]	29.1 [27.7-30.4]
Nausea/vomiting	2286	516	22.6 [20.9-24.3]	25.5 [23.5-27.6]
Diarrhoea	3048	744	24.4 [22.9-26.0]	27.7 [25.9-29.6]
Abdominal pain	2230	452	20.3 [18.7-22.0]	22.7 [20.8-24.8]
Runny nose	4327	732	16.9 [15.8-18.1]	18.7 [17.4-20.1]
Sneezing	3293	550	16.7 [15.5-18.0]	18.4 [16.9-20.0]
Blocked nose	4217	743	17.6 [16.5-18.8]	19.5 [18.2-21.0]
Sore eyes	2945	610	20.7 [19.3-22.2]	23.3 [21.6-25.1]
Loss of sense of smell	4714	2159	45.8 [44.4-47.2]	53.5 [51.8-55.2]
Loss of sense of taste	5247	2212	42.2 [40.8-43.5]	49.1 [47.5-50.7]
Sore throat	8994	1336	14.9 [14.1-15.6]	16.2 [15.3-17.1]
Hoarse voice	3376	502	14.9 [13.7-16.1]	16.2 [14.8-17.7]
Headache	10374	2128	20.5 [19.7-21.3]	23.0 [22.1-24.0]
Dizziness	3956	824	20.8 [19.6-22.1]	23.4 [21.9-25.0]
Shortness of breath	6899	1259	18.2 [17.4-19.2]	20.3 [19.2-21.4]
New persistent cough	9746	1537	15.8 [15.1-16.5]	17.3 [16.5-18.2]
Tight chest	6931	1228	17.7 [16.8-18.6]	19.7 [18.6-20.8]
Chest pain	3408	590	17.3 [16.1-18.6]	19.2 [17.7-20.7]
Fever	9128	1906	20.9 [20.1-21.7]	23.5 [22.5-24.5]
Chills	5329	1233	23.1 [22.0-24.3]	26.2 [24.8-27.6]
Difficulty sleeping	5366	930	17.3 [16.3-18.4]	19.2 [18.0-20.4]
Tiredness	12246	2626	21.4 [20.7-22.2]	24.1 [23.3-25.0]
Severe fatigue	5089	1256	24.7 [23.5-25.9]	28.0 [26.6-29.5]
Numbness/tingling	1446	358	24.8 [22.6-27.0]	28.1 [25.5-30.9]
Heavy arms/legs	5196	1173	22.6 [21.5-23.7]	25.5 [24.2-26.9]
Muscle aches	10205	2236	21.9 [21.1-22.7]	24.7 [23.8-25.7]
None of these symptoms	74	6	8.1 [3.8-16.6]	8.1 [2.9-18.3]
**Symptom severity**				
None	683	87	12.7 [10.4-15.4]	13.7 [10.9-16.9]
Mild symptoms	6071	1025	16.9 [16.0-17.8]	18.7 [17.5-19.8]
Moderate symptoms	9261	1850	20.0 [19.2-20.8]	22.4 [21.4-23.4]
Severe symptoms	3501	884	25.2 [23.8-26.7]	28.7 [27.0-30.5]
**Contact with case**				
Yes, with confirmed case	3946	742	18.8 [17.6-20.1]	21.0 [19.5-22.5]
Yes, with suspected case	5307	896	16.9 [15.9-17.9]	18.7 [17.5-19.9]
No, not to my knowledge	90655	3906	4.3 [4.2-4.4]	3.5 [3.3-3.7]
**Number of pre-existing health conditions**[‡]				
>1	22127	1097	5.0 [4.7-5.3]	4.3 [4.0-4.6]
1	28308	1558	5.5 [5.2-5.8]	4.9 [4.6-5.3]
0	49473	2889	5.8 [5.6-6.0]	5.3 [5.1-5.6]
**BMI**[$]				
Underweight (<18.5)	1236	65	5.3 [4.1-6.6]	4.6 [3.3-6.3]
Normal (18.5-24.9)	36191	1919	5.3 [5.1-5.5]	4.7 [4.4-5.0]
Overweight (25-29.9)	31818	1826	5.7 [5.5-6.0]	5.2 [4.9-5.5]
Obese (>=30)	19855	1161	5.8 [5.5-6.2]	5.4 [5.0-5.8]
**Smoking (current)**				
Yes	10635	433	4.1 [3.7-4.5]	3.2 [2.8-3.7]
No	88290	5059	5.7 [5.6-5.9]	5.2 [5.0-5.4]

[*]All estimates of prevalence adjusted for imperfect test sensitivity and specificity (see methods for details).

[†]Essential worker: List of essential workers as defined by the UK Government https://www.gov.uk/guidance/coronavirus-covid-19-getting-tested#essential-workers

[§]Other worker: Worker not working in health or social care or on the UK Government list of essential workers

[‡] Pre-existing health conditions included: organ transplant recipient, diabetes (type I or II), heart disease or heart problems, hypertension, overweight, stroke, kidney disease, liver disease, anaemia, asthma, other lung condition, cancer, condition affecting the brain and nerves, weakened immune system/reduced ability to deal with infections, depression, anxiety, psychiatric disorder, none of these

[$] Body Mass Index (BMI) formula: weight (kg) / (height [m])^2

Supplementary Table 4: Prevalence of SARS-CoV-2 IgG antibodies, adults in England, by household income and age

	Household income			
**Age**	**£0-14,999**	**£15,000-49,999**	**£50,000-149,999**	**>£150,000**
18-24	72/1162 (5.78%, CI: 4.28-7.63)	299/4989 (5.53%, CI: 4.78-6.37)	424/5720 (7.24%, CI: 6.46-8.10)	73/741 (10.18%, CI: 7.84-13.02)
25-34	82/2049 (3.13%, CI: 2.21-4.27)	736/14483 (4.44%, CI: 4.02-4.88)	1089/16625 (6.21%, CI: 5.76-6.67)	116/1174 (10.22%, CI: 8.32-12.43)
35-44	131/2800 (3.95%, CI: 3.08-4.97)	757/16309 (3.91%, CI: 3.53-4.31)	1137/22714 (4.34%, CI: 4.01-4.70)	178/2896 (5.72%, CI: 4.73-6.84)
45-54	139/3546 (3.04%, CI: 2.32-3.87)	1042/19665 (4.70%, CI: 4.33-5.09)	1328/24419 (4.87%, CI: 4.53-5.22)	256/3915 (6.19%, CI: 5.31-7.18)
55-64	215/5405 (3.11%, CI: 2.52-3.77)	1153/24155 (4.06%, CI: 3.75-4.40)	957/16910 (5.13%, CI: 4.72-5.56)	163/2094 (7.69%, CI: 6.40-9.17)
65-74	174/5503 (2.12%, CI: 1.60-2.72)	687/21050 (2.25%, CI: 1.97-2.54)	228/6147 (2.78%, CI: 2.25-3.39)	13/416 (2.08%, CI: 0.52-4.67)
75+	95/2931 (2.22%, CI: 1.52-3.07)	223/7755 (1.78%, CI: 1.36-2.25)	45/1655 (1.59%, CI: 0.77-2.67)	6/124 (4.14%, CI: 1.01-10.55)

Supplementary Table 5 Infection Fatality Ratio sensitivity analyses including care home residents and all excess deaths

Category	Population (000s)	SARS-CoV-2 prevalence % [95% CI]	Confirmed COVID-19 deaths	All excess deaths	IFR based on COVID-19 deaths % [95% CI]	IFR based on excess deaths % [95% CI]
**Total**	56,287	5.96 [5.70, 6.75]	48633	53857	1.43 [1.37, 1.49]	1.58 [1.51, 1.65]
**Sex**						
Male	27,828	6.17 [5.76, 6.59]	26787	28820	1.54 [1.43, 1.64]	1.65 [1.54, 1.76]
Female	28,459	5.75 [5.42, 6.09]	21840	25112	1.32 [1.24, 1.39]	1.51 [1.42, 1.60]
**Age**						
15-44	21,335	7.20 [6.73, 7.66]	524	550	0.03 [0.03, 0.04]	0.04 [0.03, 0.04]
45-64	14,406	6.18 [5.78, 6.58]	4657	6315	0.52 [0.49, 0.55]	0.70 [0.66, 0.75]
65-74	5,576	3.16 [2.67, 3.66]	7105	8288	3.87 [3.29, 4.46]	4.49 [3.81, 5.16]
75+	4,778	3.30 [2.53, 4.08]	36341	38779	18.71 [15.13%, 22.29]	19.72 [15.99, 23.45]

Infection fatality ratios were calculated using confirmed COVID-19 deaths (including care home residents) and using excess (all-cause) deaths. Data on COVID-19 deaths and excess all-cause deaths were obtained from PHE Fingertips report, *Excess mortality in England, week ending 17 July 2020*. https://fingertips.phe.org.uk/static-reports/mortality-surveillance/excess-mortality-in-england-week-ending-17-Jul-2020.html

Supplementary Methods

Weighting strategy

The weighting approach used rim weighting to adjust to population estimates of: age by sex; Index of Multiple Deprivation (IMD) deciles; Local Authority (LA) counts; ethnic group. The age by sex and LA counts were extracted from the ONS mid-year population estimates (1), the ethnic group counts from the Labour Force Survey (Annual Population Survey) (2), and the IMD deciles profile were derived from the original anonymous population sampling frame from NHS digital. To allow for the different sources of population estimates, the rim weighting was carried out on the proportions rather than population totals. Age was grouped into seven categories: 18 to 24; 25 to 34; 35 to 44; 45 to 54; 55 to 64; 65 to 74; 75 or older. Age and sex groups had 14 categories. The reported ethnicity was grouped into nine categories: white; mixed / multiple ethnic groups; Indian; Pakistani; Bangladeshi; Chinese; any other Asian background; black African / Caribbean / other; and any other ethnic group or missing. The rim weighting was carried out in two stages. At the first stage, the sample was weighted to LA counts and age by sex groups only. This put the sample back into the correct proportion for LAs which corrects for the disproportionate sampling and differential non-response. In the same stage, the age and sex groups were also adjusted to make sure that the final weighted profile was as close to the population as possible. The second stage of rim weighting adjusted to all four measures, using the first stage weights as the starting weights. The adjustment factor between the first and second stage weights were trimmed at the 1st and 99th percentiles to dampen the extreme weights which improves efficiency. The final weights were calculated as the first stage weights multiplied by the trimmed adjustment factor for the second stage.

Sample profile

The registration profile was monitored in relation to attributes: sex, age groups, and region which were included on the sample frame, but not others like ethnicity and occupation. As response rate exceeded expectation attempts to balance the registration profile through, for example, targeted reminders were not undertaken with weighting used to balance achieved responses from those completing the survey at the analysis stage. The age, sex and region profile of participants was broadly similar to the overall adult population profile of England. As in almost all population surveys, our study had unequal participation, with lower response among people from minority ethnic groups and in more deprived areas. We re-weighted the sample to account for such differential response.

Sampling frame, response and weighting

	Population of England profile[1, 2]	Sample profile (%)	Registered - all (%)	Registered - for kit (%)	Completed survey - unweighted (%)	Completed survey - weighted (%)
**Sex**						
Male	48.9%	49.5%	44.6%	44.2%	43.8%	48.9%
Female	51.1%	50.5%	55.4%	55.8%	56.2%	51.1%
**Age**						
18 to 24	10.9%	10.2%	6.9%	7.0%	6.6%	10.9%
25 to 34	17.3%	17.7%	13.7%	14.0%	13.4%	17.2%
35 to 44	16.1%	17.0%	16.5%	16.9%	16.8%	16.1%
45 to 54	17.5%	17.0%	19.6%	19.9%	20.3%	17.5%
55 to 64	15.0%	15.5%	19.7%	19.8%	20.4%	15.1%
65 to 74	12.6%	12.2%	15.7%	15.3%	15.8%	12.7%
75+	10.5%	10.5%	7.9%	7.0%	6.6%	10.6%
**Region**						
North East	4.8%	3.7%	3.7%	3.6%	3.6%	4.8%
North West	13.0%	12.4%	12.2%	12.2%	12.0%	13.0%
Yorkshire & Humber	9.8%	6.3%	6.5%	6.5%	6.5%	9.8%
East Midlands	8.7%	11.9%	12.6%	12.6%	12.6%	8.7%
West Midlands	10.5%	9.4%	9.6%	9.6%	9.6%	10.4%
East of England	11.1%	13.3%	14.4%	14.4%	14.5%	11.1%
London	15.6%	15.3%	9.9%	9.9%	9.7%	15.6%
South East	16.3%	19.5%	21.7%	21.7%	22.0%	16.3%
South West	10.2%	8.2%	9.4%	9.4%	9.5%	10.3%
**IMD quintile**						
1	19.8%	17.4%	10.9%	10.8%	10.2%	19.4%
2	20.9%	20.5%	16.4%	16.4%	16.0%	20.9%
3	20.4%	21.2%	21.5%	21.5%	21.6%	20.5%
4	19.7%	20.7%	24.4%	24.5%	24.8%	19.9%
5	19.1%	20.2%	26.7%	26.8%	27.4%	19.3%
**Ethnicity**						
White	86.3%	NA	92.2%	92.2%	92.7%	87.0%
Mixed/Multiple ethnic groups	1.1%	NA	1.4%	1.4%	1.4%	1.1%
Indian	2.9%	NA	2.0%	2.0%	1.9%	2.9%
Pakistani	1.9%	NA	0.6%	0.6%	0.5%	1.7%
Bangladeshi	0.8%	NA	0.2%	0.2%	0.2%	0.7%
Chinese	0.5%	NA	0.4%	0.4%	0.4%	0.5%
Any other Asian background	1.2%	NA	0.8%	0.8%	0.7%	1.3%
Black/African/Caribbean/Black British	3.3%	NA	1.1%	1.1%	0.9%	2.8%
Other ethnic group / Don’t know	1.9%	NA	1.4%	1.4%	1.3%	1.8%

Study response rates

	Round 1 (Jun 20–Jul 13)	% of all invited
Invitation letters sent*	314,996	
Registered[ł]	126,143	40.0%
Requested test kit	121,976	38.7%
Completed survey	109,076	34.6%
Attempted test	107,640	34.2%
Completed test	105,651	33.5%
Valid test	99,908	31.7%

*The number of invitations sent out was based on an assumed response rate of 38%.
[ł] Registration was stopped when target reached approx. 120,000 to ensure sufficient test kits would be available, and therefore overall response rate is artificially capped.

Regions of England

England is the largest of the four geographical areas making up the United Kingdom, the other three being devolved nations of Northern Ireland, Scotland and Wales. England is further divided into nine administrative regions: North East, North West, Yorkshire, East Midlands, West Midlands, East of England, London, South East, South West. The regions are the highest tier of sub-national division in England and are used for statistical and some administrative purposes. In the table below some key demographic characteristics of the regions are provided.

Selected characteristics of Regions of England

	Population[(1)]	Area (km^2)[(1)]	Population density (per km^2)[(1)]	Median gross annual earnings (£) in 2019[(2)]	% population claiming income support in Aug 2019[(3)] (n)
North East	2,669,941	8,579	311	27,187	0.89 (23,711)
North West	7,341,196	14,108	520	28,137	0.63 (46,409)
Yorkshire and Humber	5,502,967	15,405	357	27,835	0.70 (38,272)
East Midlands	4,835,928	15,625	309	28,000	0.56 (26,896)
West Midlands	5,934,037	12,998	457	28,536	0.69 (40,848)
East of England	6,236,072	19,116	326	30,345	0.45 (28,363)
London	8,961,989	1,572	5,701	38,992	0.58 (52,266)
South East	9,180,135	19,072	481	32,120	0.42 (38,664)
South West	5,624,696	23,836	236	28,654	0.39 (21,721)

[1] Office_for_National_Statistics. Population estimates for the UK, England and Wales, Scotland and Northern Ireland: mid-2019 2020 [Available from: https://www.ons.gov.uk/releases/populationestimatesfortheukenglandandwalesscotlandandnorthernirelandmid2019

[2] Office_for_National_Statistics. Employee earnings in the UK: 2019 2019 [Available from: https://www.ons.gov.uk/releases/employeeearningsintheuk2019.

[3] Department_for_Work_and_Pensions. DWP benefits statistics 2019 [Available from: https://www.gov.uk/government/collections/dwp-statistical-summaries.

Map showing Regions of England with prevalence of SARS-CoV-2 antibody

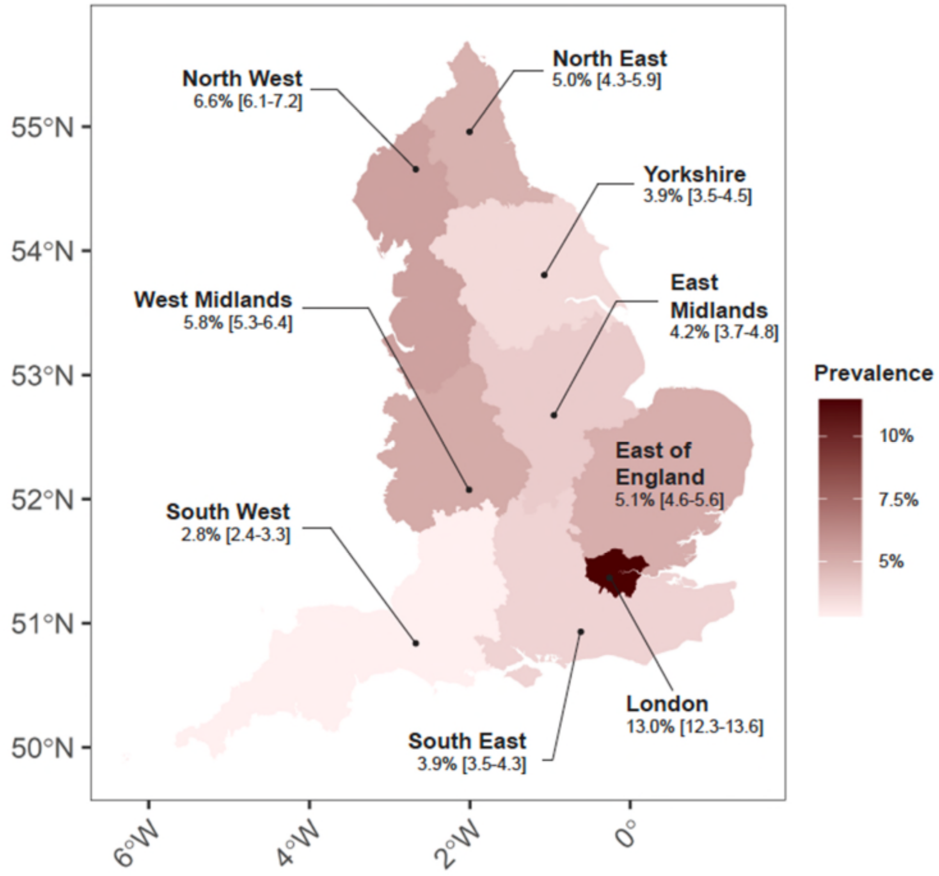

Source: Map generated from Office for National Statistics licensed under the Open Government Licence v.3.0. Contains OS data © Crown copyright and database right [2020]. Source data in Supplementary Data 1.

Missing data

In the final data set used for analysis, 8 variables had missing data that was not a logical consequence of skip patterns in the questionnaire as shown in the plot below. The only variable with large amount of missing data was BMI, which is computed from self-reported weight (11514 missing) and height (4741 missing) leading to 11% with a missing BMI.

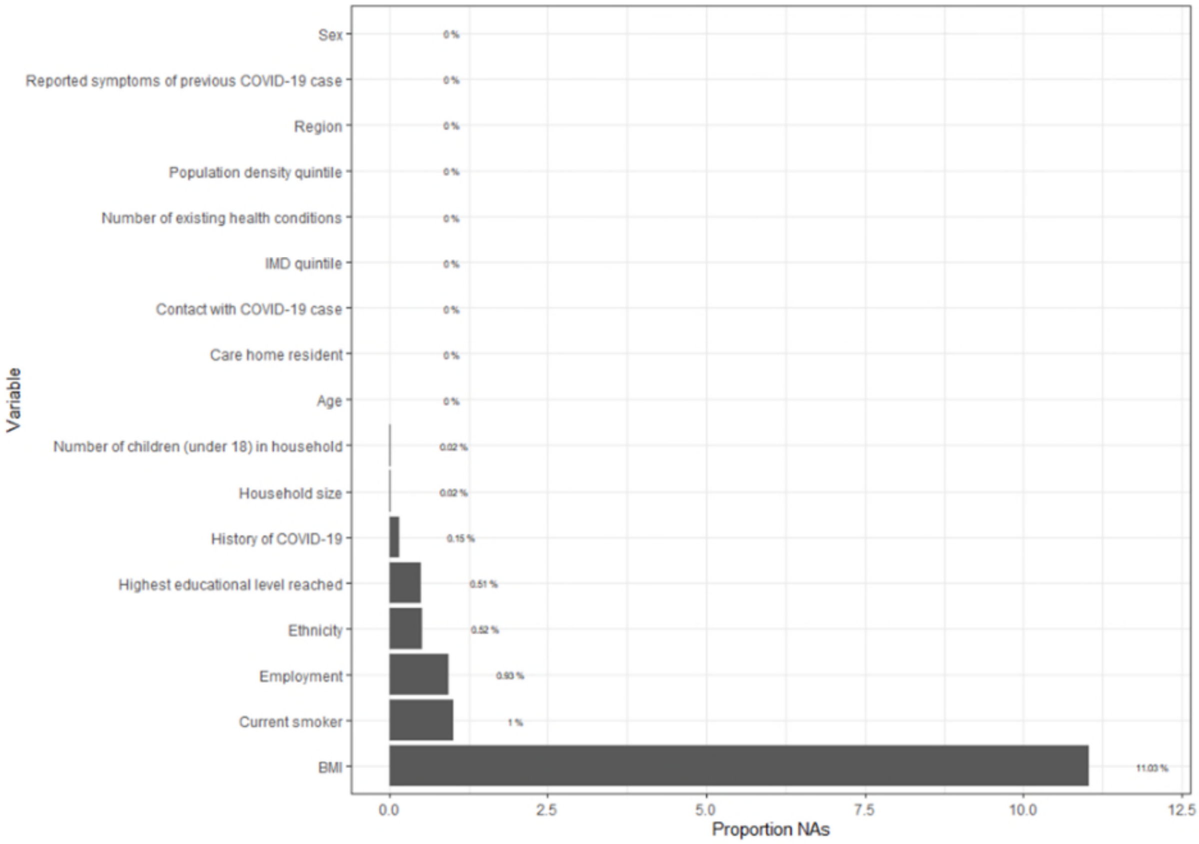

Supplement: Supplementary file 1 — Supplementary Information [file 41467_2021_21237_MOESM1_ESM.pdf]
