# Supplementary material for: SARS-CoV-2 antibody prevalence in England following the first peak of the pandemic
Source: Nat Commun. 2021 Feb 10;12:905. doi: 10.1038/s41467-021-21237-w (PMC7876103; doi:10.1038/s41467-021-21237-w)
Supplement: Supplementary file 3 — Reporting Summary [file 41467_2021_21237_MOESM3_ESM.pdf]

nature research

Corresponding author(s): Helen Ward, Paul Elliott

Last updated by author(s): Jan 5, 2021

Reporting Summary

Nature Research wishes to improve the reproducibility of the work that we publish. This form provides structure for consistency and transparency in reporting. For further information on Nature Research policies, see our Editorial Policies and the Editorial Policy Checklist.

Statistics

For all statistical analyses, confirm that the following items are present in the figure legend, table legend, main text, or Methods section.

n/a	Confirmed	
☐	☒	The exact sample size (*n*) for each experimental group/condition, given as a discrete number and unit of measurement
☒	☐	A statement on whether measurements were taken from distinct samples or whether the same sample was measured repeatedly
☒	☐	The statistical test(s) used AND whether they are one- or two-sided<br>*Only common tests should be described solely by name; describe more complex techniques in the Methods section.*
☐	☒	A description of all covariates tested
☐	☒	A description of any assumptions or corrections, such as tests of normality and adjustment for multiple comparisons
☐	☒	A full description of the statistical parameters including central tendency (e.g. means) or other basic estimates (e.g. regression coefficient) AND variation (e.g. standard deviation) or associated estimates of uncertainty (e.g. confidence intervals)
☒	☐	For null hypothesis testing, the test statistic (e.g. *F*, *t*, *r*) with confidence intervals, effect sizes, degrees of freedom and *P* value noted<br>*Give P values as exact values whenever suitable.*
☒	☐	For Bayesian analysis, information on the choice of priors and Markov chain Monte Carlo settings
☒	☐	For hierarchical and complex designs, identification of the appropriate level for tests and full reporting of outcomes
☒	☐	Estimates of effect sizes (e.g. Cohen's *d*, Pearson's *r*), indicating how they were calculated

Our web collection on statistics for biologists contains articles on many of the points above.

Software and code

Policy information about availability of computer code

Data collection	All data collection for the REACT2 study is captured with Questback (Spring 2020 installation).
Data analysis	All statistical analysis and visualisation was conducted with R version 4.0.0

For manuscripts utilizing custom algorithms or software that are central to the research but not yet described in published literature, software must be made available to editors and reviewers. We strongly encourage code deposition in a community repository (e.g. GitHub). See the Nature Research guidelines for submitting code & software for further information.

Data

Policy information about availability of data

All manuscripts must include a data availability statement. This statement should provide the following information, where applicable:

- Accession codes, unique identifiers, or web links for publicly available datasets
- A list of figures that have associated raw data
- A description of any restrictions on data availability

The aggregated tables generated during this study are provided as supplementary data files

Field-specific reporting

Behavioural & social sciences study design

All studies must disclose on these points even when the disclosure is negative.

Study description	A cross sectional quantitative survey to investigate COVID-19 seroprevalence in the adult England population.
Research sample	We obtained a random population sample of adults in England, using the National Health Service (NHS) patient list, which includes name, address, age and sex of everyone registered with a general practitioner (almost the entire population). Personalized invitations were sent via post to 315,000 individuals aged 18 years and above to achieve similar numbers in each of 315 lower-tier local authority areas. The initial sample was designed to be representative of the adult population of England and responses were weighted to account for non-response.
Sampling strategy	random sample as above. Sample size determination has been published in the protocol which states: We are aiming for precision in our estimates of prevalence at local tier local authority level. Estimates will be adjusted for test validity as measured in the laboratory-based evaluation sub-study of LFIAs. Based on a conservative sensitivity of 72% and overall prevalence of 7% we estimated the lower and upper bounds 95% binomial confidence intervals: with 100,000, CI 5.05 9.63, with 150,000 5.36 9.09.
Data collection	Data were collected by participants completing an online (or phone if requested) registration survey and then a second survey when they completed a finger-prick test. Researchers were no blinded to experimental condition/hypothesis.
Timing	Data were collected in a single collection period between 20 Jun and 13 July 2020.
Data exclusions	We used complete case analysis.
Non-participation	The overall response rate (all invited to all completing survey and attempting test) was 34.2%. Details are provided in supplementary material.
Randomization	Participants were not allocated to experimental groups.

Reporting for specific materials, systems and methods

We require information from authors about some types of materials, experimental systems and methods used in many studies. Here, indicate whether each material, system or method listed is relevant to your study. If you are not sure if a list item applies to your research, read the appropriate section before selecting a response.

Materials & experimental systems

n/a	Involved in the study	
☒	☐	Antibodies
☒	☐	Eukaryotic cell lines
☒	☐	Palaeontology and archaeology
☒	☐	Animals and other organisms
☐	☒	Human research participants
☒	☐	Clinical data
☒	☐	Dual use research of concern

Methods

n/a	Involved in the study	
☒	☐	ChIP-seq
☒	☐	Flow cytometry
☒	☐	MRI-based neuroimaging

Human research participants

Policy information about studies involving human research participants

Population characteristics	See above
Recruitment	We obtained a random population sample of adults in England, using the National Health Service (NHS) patient list, which I includes name, address, age and sex of everyone registered with a general practitioner (almost the entire population). Personalized invitations were sent via post to 315,000 individuals aged 18 years and above. We quantify differential response rates by age, sex, ethnicity and deprivation which are similar to those regularly observed in population surveys. We adjust for this in analysis through weighting the sample.
Ethics oversight	We obtained research ethics approval from the South Central-Berkshire B Research Ethics Committee (IRAS ID: 283787), and MHRA approval for use of the LFIA for research purposes only, and participants provided informed consent.

Note that full information on the approval of the study protocol must also be provided in the manuscript.
